# Progress in Phototherapy and Electro-Therapeutics

**Published:** 1903-10-03

**Authors:** 


*(To be continued.)*

### Progress in Phototherapy and Electro-Therapeutics.

(*Continued from Vol. XXXIV., page* 453.)

Light.—Finsen's statistics[8] show that 804 cases of lupus have been under treatment. Of these—

I. 412 are regarded as cured: (*a*) Free from relapses in 2–6 years=124; (*b*) Time of observation less than two years=288.

II. Almost cured (only insignificant traces of the disease persisting)=192.

III. Under treatment=117: (*a*) Improved or partly cured, 91; (*b*) Little influenced by treatment, 26.

IV. Incompletely cured=83: (*a*) Unsatisfactory results, 16; (*b*) Dead (31), or suffering from other disease, 44; (*c*) External circumstances, 23.

That is 94 per cent. have been greatly benefited, if not entirely cured; and 6 per cent. have proved unsatisfactory.

Leredde and Pautrier[9] find good results with the Lortet-Genoud lamp, but require an hour's application to get sufficient penetration. They condemn the iron electrode lamps as being far too superficial. They consider that light holds its own with any known methods of treatment of lupus and is better, with the present methods of administration, than the *x*-rays. With lupus erythematosus they have had good results with the Finsen lamp. Out of 23 cases, 13 have been cured or almost so, and 3 markedly improved. They recommend light for rosacea, sycosis, tuberculides, and alopecia.

Busck[10] contends the statement of Kromayer's that the Dermo lamp is 30 to 40 times more powerful than Finsen's, judging by the reaction of the skin. He has found this by many experiments not to be the case. The bacillus prodigiosus is unaffected by an exposure of 5 minutes to the Dermo lamp through a screen of methylene blue 8 in 100,000, while the 70 amp. carbon lamp kills it in less than one second. Silver chloride paper protected by two bloodless rabbit's ears is totally blackened in 3 to 4 seconds with 80 amp. lamp, while the iron lamp with 14 amps. takes 3 minutes. The chloride paper is hardly affected by light rays at the less refrangible end of the spectrum. This proves then that Kromayer's apparatus works with roughly $\frac{1}{60}$th of the blue violet rays possessed by the Finsen lamp.

The members of the Institute of Experimental Medicine at St. Petersburg are pleased with the experiments they have made to test Finsen's method.[11] In 12 months 44 patients were treated, and these included cases of lupus vulgaris, lupus erythematosus, verrucose tuberculosis of the skin, rodent ulcer, vascular nævus, and trophic ulcer. Eight of the 26 cases of lupus vulgaris were cured; 1 out of 9 of the erythematosus, 3 being decidedly improved. In the 5 patients with nævus, marked improvement was observed when patients discontinued treatment. Some affections of the mucous membranes were also successfully treated.

The action of light upon alopecia areata is not yet understood. Schmidt,[12] to test its action on the growth of hair, depilated two equal patches on two guinea-pigs. He placed one in a small box exposed to ordinary sunlight and the other in a box with a window of red glass, which was only transparent to red, orange, yellow and green: the blue, violet, and ultra-violet rays being cut off. He could detect no difference in the two, except perhaps one possibly favourable to the red rays. Two cases of alopecia areata were treated with the Finsen lamp, one with a compressor and quarter-hour exposures, which got well after seven exposures, and the other without a compressor, the lamp being one metre from the scalp, and in this the hair fell out all over the scalp. These experiments he does not regard as conclusive.

Having observed that a case of lupus erythematosus was always improved after exposure to the glare from the water when fishing in the Highlands, Norman Walker[13] has tried directing the light of London Hospital lamps on such cases at the distance of a foot or so. Exposures of 20 to 30 minutes were made daily or on alternate days, six or eight exposures being made before any result was apparent, but when improvement began it was rapid. In some cases results were brilliantly successful, all the cases having shown improvement. Large areas of disease disappeared, and in some cases more thoroughly than under any other method. Latterly he has used adrenalin chloride 1-4,000 applications, in addition to the light, and it has seemed to aid the cure.

Carrying out the red-light treatment for small-pox as recommended by Finsen, Schamberg[14] has been disappointed in his results. In two cases the method failed absolutely to exercise any modifying influence. One patient died, and the other was left with very much scarring.

Minin[15] tells us of the analgesic properties of coloured light, and Tsiechauski[16] reports 8 cases of tubercular joint affections, five in children and three in adults, treated successfully with a 12,000 candle-power lamp (35-40 V and 80-120 amps.). He states that heat and light cure in a painless manner, and replace other measures of treatment usually employed. The value of light can be increased, according to Gamlen,[17] by the use of vaseline.

**X-rays.**—In Coley's opinion, the $x$-rays have an inhibitory action on all forms of malignant tumours,[18] but which varieties are most susceptible cannot yet be particularised owing to insufficiency of cases. At present, primary sarcomata of lymph glands and superficial epithelioma seem to yield most readily, yet it is too recent to state that any case has been *cured* by the rays; and at present it is not warrantable to treat an operable primary case. In several cases of inoperable round-celled sarcomata, where toxins had been tried without result, $x$-rays caused entire disappearance of the tumours, though recurrence occurred speedily; yet in spindle-celled sarcoma of the kind most amenable to toxin treatment $x$-rays had a very small effect. In the round-celled variety, where toxins were useless, the rays seem to have had the best results, and there is reason to believe that the combined treatment will give better results than either used alone.

Forty-seven cases of cancer have been treated with the $x$-rays by C. W. Allen,[19] including 30 more or less dermatological cancers, 10 of the breast, one rectal, one uterine, one glands and tissues of neck, three sarcomata, and one doubtful sarcoma. The dermatological cases included a case of xeroderma pigmentosum. His results show five ending fatally, 25 discharged cured, three ceased treatment improved, five ceased treatment unimproved, and nine improved and under treatment. His impressions are that the rays have a decided therapeutic power, but a possibility of marked injurious results. Sometimes the effect of treatment is shown on the heart, lungs, and other internal organs, symptoms pointing to too rapid absorption of disintegration products. Metastases occur at times in grave forms of cancer more rapidly than we are accustomed to see in patients not so treated. There is a possibility also of cancer being produced by the rays, as in the case of the tube-maker who developed carcinoma on an $x$-ray cicatrix of the forearm, and now, as is published this month,[20] where an $x$-ray ulcer has become epitheliomatous, requiring amputation of the limb. The method is not to be relied upon in all forms of cancer; in nodules, wart-like and dry growths upon the skin, other means of removal, preferably arsenical paste, should be tried and the rays employed subsequently.

In order to try and obtain statistics Brockman[21] has written to numerous surgeons, laryngologists, and dermatologists for their experiences in treating disease with the $x$-rays. His results he gives in the following figures:—Of 44 sarcomata 10 cases are apparently cured, 1 improved, and 17 not improved; some of these latter were not long under treatment, or were bad cases, all cases were inoperable. Of 52 superficial carcinomata, 32 are reported healed or apparently cured, 5 improved, pain reduced, and tumour decreased in size, 10 not improved, and 6 now under treatment. Of deep carcinomata, 4 "cured," 5 improved, and 15 failures. He has had no reports on tuberculous laryngitis, but in another place Delavan[22] reports improvement in a case of laryngeal cancer with the rays—the patient, however, died of intercurrent Bright's disease. Of 41 cases of tuberculous glands, 10 are apparently cured, 4 improved, and 27 are failures.

Several cases of splenic leukæmia are reported as apparently cured. Deep-seated growths do not seem to yield to the rays. At the Massachusetts Hospital[23] though many cases have been faithfully exposed, few have essentially improved and none has been cured. Codman considers that the rays have their place in relieving pain, allowing of gain in body weight, with shrinkage, softening and breaking down in the tumour. In epithelioma about 100 cases have healed or are healing. In a favourable case of cancer of the tongue, which had apparently been removed entirely, Codman started $x$-ray treatment as prophylaxis, keeping just short of troublesome dermatitis. He treated the case from May to September, by which time the disease had so far returned in the mouth and neck as to make the treatment a mere nuisance. In a case of cancer of breast (unoperated) under treatment, keeping the skin constantly exfoliating and at times blistered, the tumour seemed to remain stationary. Seven recurrent nodules in a breast case disappeared after five months' treatment. No success with two cancers of the penis. A round-celled sarcoma of the sterno-clavicular region, inoperable, 5 in. $\times 2\frac{1}{2}$ yielded to the $x$-rays; but other cases of sarcoma have resisted treatment. Codman thinks that the good effect of the rays as shown by the disappearance of psoriasis, chronic eczema, lupus and superficial epithelioma is due to stimulation of nutrition rather than destruction of malignant tissue, because:—(1) The effect is delayed sometimes for weeks; (2) the primary effect is increased stimulation of the skin as evidenced by hyperæmia and exfoliation; (3) in one case of massive malignant disease of the breast no destruction of cells was found; degeneration in the interior in such cases not treated by the rays is often found; (4) the appearance of the shrunken cancer-cells surrounded by granulation tissue might be explained by activity of tissue; (5) in a case of varicose eczema of the leg, the eczema not only disappeared but the leg shrank to normal size; (6) the good effects are mainly observed in those diseases characterised by a disordered or indolent local nutrition, which nature when stimulated had more chance to throw off; (7) nature is more likely to aid in just those diseases where $x$-rays prove of service; in cancer, nature is impotent to check great malignancy; (8) good and bad effects occur chiefly in skin where the nutrition is not so stable as in underlying parts.

With regard to deep seated malignant growths, Richmond[24] has treated a case of supposed sarcoma of the kidney, and it has disappeared under this treatment. The patient was a married woman, æt. 40, with a rapidly-growing, deep-seated tumour in left hypochondrium, dulness to percussion reaching posteriorly as high as the eighth rib; it was so rapidly growing that it was thought inadvisable to operate. Night sweats ensued, weight ran down, and menstruation ceased. The circumference at the ensiform cartilage was two and a half to three inches greater than normal, and the tumour extended to the crest of ilium. $X$-rays were administered 15 minutes for 19 consecutive days when the temperature fell nearly to normal, the night sweats lessened, the tumour was softer and had ceased to grow. The patient was then removed to the hospital for more efficient $x$-ray treatment; 12 weeks after the commencement of treatment the growth could not be detected.

[8] Brit. Jour. of Dermatol., May. [9] Ibid. [10] Ibid., Aug. [11] Brit. Med. Jour., June 13. [12] Brit. Jour. of Dermatol., June. [15] Brit. Med. Jour., June 27. [14] Ibid., June 20. [15] Ibid., June 13. [16] Ibid. [17] Ibid., June 6. [18] Ibid, July 18. [19] Ibid., May 2. [20] Brit. Jour. of Dermatol., Aug. (Münch. Med. Wochensch., Nov. 24, 1903). [21] Brit. Med. Jour., June 6. [22] Ibid. [25] Johns Hopkins Hosp. Mag., May. [24] Brit. Med. Jour., July 4.

*(To be continued.)*

